# Genito-Urinary and Syphilitic Diseases

**Published:** 1898-11

**Authors:** Byron H. Daggett

**Affiliations:** Buffalo, N. Y.


### GENITO-URINARY AND SYPHILITIC DISEASES.

Conducted by BYRON H. DAGGETT, M. D., Buffalo, N. Y.

### LUMBAR NEPHROPEXY WITHOUT SUTURING.

SENN (*Denver Med. Times*, December, 1897,) states that nephropexy is a legitimate surgical procedure in all cases that can be established ; that the displaced kidney is the direct cause of the manifold symptoms which such a condition often produces. In nearly all the operations, the kidney is sutured to the muscular structures, exposed by the incision and the lower border of the last rib. Four sutures are usually used and are passed through the capsule and more or less of the parenchyma of the kidney. The posterior surface is exposed by incising a portion of the capsule, or by reflecting it. It was demonstrated that the fibrous capsule of the kidney would proliferate its kind, under mechanical irritation and aseptic conditions, and thereby adhere to its neighbor.

Nicolandi incises the fibrous capsule sufficient to get a flap without disturbing the parenchyma. All sutures fail to maintain the kidney in the desired location a sufficient length of time to effect permanent fixation. The objections to including the kidney substance is that it is friable and that pathologic conditions result from the presence of sutures in the parenchyma.

Senn dispenses with the use of sutures ; he extensively incises the pararenal fat, scarifies the capsule ; supports the kidney temporarily by a strip of iodoform gauze and tamponade ; maintains prolonged dorsal recumbent position and abdominal compression over the kidney. Free incision of the adipose capsule over the entire posterior surface of the kidney forms a gutter for lodging the kidney, from which it is difficult to dislodge it as long as the patient remains upon his back.

Senn exposes the kidney by Simon's vertical lumbar incision. The adipose capsule is freely incised and reflected, the fibrous capsule is scarified with a large needle. The capsule of the kidney is then grasped at the lower pole of that organ with a vulsellum and brought well forward into the wound. The lower end of the kidney is freed from its capsule and four layers of gauze, one inch wide, are placed underneath the end of the organ and tied over the tampon. The patient is placed upon the back and a compress placed over the kidney, below the costal cartilage. The pelvis is slightly elevated and the patient kept in bed, resting in the dorsal recumbent position upon the wound side for at least four weeks. The tampon is removed at the end of five or six days after the operation and the whole wound will be found paved with rigorous granulations ; the edges of the wound are now drawn together by strips of adhesive plaster and held by a gauze roller. Dr. Senn closes his article as follows : The retention of the kidney in its normal location by this method affords not only a firm support by permanently fixing the lower end in the lower angle of the external incision, and by securing broad surface adhesions, but the oblique angle in which it is anchored adds another mechanical condition, calculated to maintain the organ in position, will also tend to correct flexion of the ureter, if such exists at the time the operation is performed. The immediate and remote results obtained by this method of operating in the last four cases have proved so satisfactory that I am not disposed to return to suturing again, and strongly recommend this method of performing nephropexy for further trial by the profession.

---

### EXPERIENCES IN THE TREATMENT OF SYPHILIS.

KRATOSZYNER (*Pacific Med. Jour.*, San Francisco, Cal.) states that rapid strides have been made in the pathology of syphilis, but its therapy has lagged in the race. For centuries mercury has been the principal remedy used in the treatment of syphilis, although repeatedly abandoned only to be taken up again. Various opinions have been expressed regarding the curability of this disease. Bärensprung said there is no cure. Kaposi, that it is not only curable, but of all infectious diseases is the one in which the best cure can be affected. It is safe to say that treatment at the right time, by the best methods and remedies, managed by an energetic physician, who is well informed as to the pathology of the disease will prevent much harm to the patient and protect the community.

The best remedy is mercury. The theory of the anti-mercurialists is not proven. Mercury is accepted by all careful observers in all civilised countries as the best curative remedy for syphilis. It never produces any lesions which have any connection with those produced by syphilis.

Engelstead stated that out of 7,424 cases of constitutional syphilis, 493 cases of severe destructive lesions occurred in patients who had never had any mercurial treatment. How many more would have occurred had not their maladies been modified by a previous mercurial course? The iodides are very valuable remedies in the advanced stages of the lesion. J. Hutchison says that the combination of mercury and iodine acts more irregularly, is more liable to purge or even to salivate unexpectedly than when used singly or uncombined. Very large doses of the iodines are often used. Keyes has given two and a half ounces each day. Joseph states that ten grains, given three times daily, will cure all the later manifestations of this disease.

In the second stage mercury is the best remedy. There are three methods of administering mercury into the system, viz.: by the mouth, hypodermic injection and by inunction. In severe cases internal administration is unsafe, because its effects are slow and uncertain. By this method it is impossible to control the amount of mercury that is absorbed, especially if gastro-enteritis is induced. Its use by injection is objectionable on account of the pain it causes, as well as infiltration about the point of injection; not only this, but persistent stomatitis is often produced.

---

### EVERSION OF THE TUNICA VAGINALIS FOR THE CURE OF HYDROCELE.

JABOULAY devised and first practised this procedure in 1893. An incision three inches long is made in the anterior and dependent portion of the scrotum down to and exposing the tunica vaginalis. The tunica is enucleated in the greater part of its extent and the mass turned out of its scrotal covering. The tunica is then opened, the testicle is turned out by eversion and the parietal walls of this membrane are cut away for a third or more of their extent.

Dolon in the *Lyon Medical*, for November 28, 1897, recommended that the remaining portion of the tunica be turned back and stitched together with catgut sutures behind the testicle. The testicle is then replaced within the scrotum and the scrotal wound closed by silver sutures and a drain left in for two days. There remains after this operation a small insensitive mass formed by the everted tunica covering the sphincter cord and the epididimis.

M. Barral, in a thesis upon this subject, says: It is ideal in its simplicity; is very easy to perform; it reduces traumatism to the minimum; it requires but few simple instruments; if asepsis is secured, there is no suppuration; the cure is radical; relapse is impossible; the patient is able to walk sooner than after other operations.

---

LOCAL ANESTHESIA.—Guaiacol in solution in oil has been used by Lucas Championniere as a local anesthetic, especially for the turbinals and for the removal of polypi in the nose and ear. Anesthesia is obtained in ten minutes. Eucain is still used for operations on the nose, throat and ear. It is less poisonous than is cocain and is used in a solution of 5 to 8 per cent.—*Cleveland Journal of Medicine.*

